# Human milk sampling should be standardized if the adequacy of HM nutrients intake is assessed

**DOI:** 10.1111/mcn.13283

**Published:** 2021-12-25

**Authors:** Agnieszka Bzikowska‐Jura

**Affiliations:** ^1^ Department of Medical Biology Medical University of Warsaw Warsaw Poland

Dear Editor,

Recently, Dumrongwongsiri et al. (2021) investigated the adequacy of iron and zinc intakes among exclusively breastfed infants during the first 4 months of their life. The authors concluded that inadequate zinc intake was found in 14.5% and 40% of infants at 2 and 4 months, respectively, whereas daily iron intake was adequate at both ages.

Considering many factors affecting HM composition, the procedure of milk sampling should be standardized, and in my opinion, the description of methodological section concerning this issue is insufficient. Although the authors indicated that to avoid within‐feed variation, mothers were asked to empty one breast by using electric breast pump, the protocol of the study (Dumrongwongsiri et al., 2020) did not provide information about time of the day the milk was collected (was it completely arbitrary?) and whether the milk collection was dependent on the time of feeding the infant from the second breast (immediately after feeding the baby or independently?). As it was previously observed (Shashiraj et al., 2006; Silvestre et al., 2000), some of the divergencies between the findings for HM iron concentration may be related to the fact that the higher concentration of this mineral is reported in the evening and at the night‐time. The confirmation for this observation is the latest study by Bilston‐John et al. (2021) who observed 24 h variation for HM iron concentration resulting in significantly lower daily intake when morning, hindmilk samples were analysed. Considering that, to minimize possible circadian influences on HM composition and therefore to ensure proper estimation of HM nutrients intake, the analysis of HM should be based on 24 h collection. Even if suggested procedure is not possible to perform, HM sampling for each study should be cautiously planned and standardized (e.g., morning single collection after feeding the baby, full emptying of the breast). Without standardization, comparing results and drawing conclusions are very limited.

## CONFLICTS OF INTEREST

The authors declare that they have no conflicts of interest.
